# The association between coagulation function and prognosis in patients with sepsis: a meta-analysis of predictive performance introduction

**DOI:** 10.3389/fmed.2025.1706082

**Published:** 2025-12-18

**Authors:** Chao Li, Wei Huang, Xinxuan Han, Haoran Li, Xianyun Qin

**Affiliations:** 1 Department of Emergency, The 945th Hospital of the Joint Logistics Support Force of the Chinese People’s Liberation Army, Ya’an, China; 2 General Surgery, The 945th Hospital of the Joint Logistics Support Force of the Chinese People’s Liberation Army, Ya’an, China; 3 Department of Orthopaedics, The 945th Hospital of the Joint Logistics Support Force of the Chinese People’s Liberation Army, Ya’an, China

**Keywords:** sepsis, meta-analysis, prognosis assessment, coagulation function, biomarker

## Abstract

**Background:**

Sepsis, a leading cause of mortality in intensive care units, is associated with coagulation dysfunction, a key pathological feature linked to multi-organ failure. However, the prognostic value of coagulation markers remains heterogeneous across studies.

**Objective:**

This meta-analysis aimed to evaluate the association between coagulation parameters and clinical outcomes in sepsis patients, focusing on their predictive performance.

**Methods:**

Following PRISMA guidelines, nine studies (*n* = 1954) were included from PubMed, Embase, Cochrane Library, and Web of Science. The associations of coagulation markers (APTT, PT, D-dimer, fibrinogen, INR) and SOFA scores with the outcome were quantified using pooled odds ratios (ORs) with 95% confidence intervals. Diagnostic accuracy was summarized by the area under the curve (AUC). Heterogeneity was assessed using the *I*^2^ statistic, and a random-effects model was employed to account for between-study variation. The robustness of the findings was evaluated through sensitivity analyses.

**Results:**

Fibrinogen showed a significant inverse correlation with mortality [OR = 0.76, 95% CI (0.59–0.97)], while INR demonstrated moderate predictive ability (AUC = 0.68). APTT and PT had non-significant ORs but moderate AUCs (0.75 and 0.75, respectively). D-dimer and SOFA score ORs were non-significant, though SOFA’s AUC indicated good prognostic utility. High heterogeneity (*I*^2^ > 74%) and sensitivity to individual studies were noted.

**Conclusion:**

Specific coagulation markers, notably fibrinogen and INR, may aid in sepsis prognosis assessment, but variability across studies limits generalizability. Further high-quality studies are warranted to validate these findings.

**Systematic review registration:**

https://www.crd.york.ac.uk/PROSPERO/view/CRD420251049209, identifier CRD420251049209.

## 1 Introduction

Sepsis is one of the leading causes of mortality worldwide, especially in intensive care units (ICUs), where its high incidence and mortality rates lead to a significant disease burden (1). The pathogenesis of sepsis is complex, primarily involving an exaggerated immune response triggered by infection, systemic inflammatory response, and microvascular dysfunction, which ultimately leads to multi-organ failure (2). Although early diagnosis and timely treatment can significantly improve the prognosis of some patients, a considerable number of patients still fail to benefit due to the inability to identify high-risk groups in a timely manner or worsening of their condition, ultimately leading to ineffective control of the disease. Therefore, identifying biomarkers that can accurately assess the severity and prognosis of sepsis, particularly clinical indicators of coagulation dysfunction, has become an important focus in current sepsis research.

Coagulation dysfunction is a core feature of the pathological process in sepsis (3). The imbalance between the coagulation and fibrinolysis systems is considered one of the key factors in the multi-organ failure induced by sepsis (4). Sepsis activates widespread coagulation responses, especially the extrinsic and intrinsic coagulation pathways and the fibrinolysis system, which may ultimately lead to life-threatening complications such as disseminated intravascular coagulation (DIC) (5). Prothrombin time (PT) and activated partial thromboplastin time (APTT) are commonly used coagulation tests that reflect coagulation factor consumption in sepsis patients (6), with prolonged values closely associated with the course of sepsis, organ failure, and mortality risk. The international normalized ratio (INR), a standardized measure of PT, is typically elevated when there is coagulation factor deficiency or suppressed coagulation function, which is also commonly observed in late-stage sepsis (6). Additionally, D-dimer, a product generated during fibrinolysis, is frequently elevated in sepsis patients, indicating excessive coagulation activation and fibrinolysis inhibition (6, 7). Fibrinogen (Fib) is another coagulation marker reflecting acute-phase responses. Its elevated levels are common in the early stages of sepsis, while a decrease in Fib as the disease progresses and coagulation factors are consumed may indicate more severe coagulation dysfunction (4). Although the roles of these coagulation markers in sepsis have been confirmed in several independent studies, there remains significant heterogeneity in the comprehensive evaluation of their prognostic value and their interrelationships in sepsis prognosis.

Currently, commonly used prognostic tools for sepsis, such as the SOFA score, can reflect organ dysfunction to some extent, but their sensitivity to coagulation dysfunction is relatively weak (8). Therefore, combining coagulation function parameters with traditional scoring systems to form a more accurate multi-parameter assessment model holds important clinical significance. In recent years, integrated scoring systems such as the ISTH, KSTH, and JAAM criteria have been increasingly applied to evaluate sepsis-associated coagulopathy (9). These frameworks have enhanced diagnostic consistency and prognostic precision. Nevertheless, the individual coagulation parameters that constitute the foundation of these scoring systems continue to provide essential information for clinical assessment, particularly in the early stages of sepsis or in resource-limited settings where comprehensive scoring is not always feasible. Although some coagulation markers have been used individually to predict sepsis prognosis, the lack of systematic evaluation of the combined predictive ability of these markers, due to differences in sample size, study design, and measurement methods, remains a significant gap in the literature (10).

This study aims to systematically evaluate the correlation between coagulation function markers and clinical outcomes in sepsis patients through meta-analysis, further exploring the clinical predictive value of different coagulation markers in early sepsis assessment. By quantifying the relationship between coagulation dysfunction and the prognosis of sepsis patients, this study hopes to provide more comprehensive and precise prognostic assessment tools for clinical practice and offer evidence-based support for the precise management of sepsis.

## 2 Materials and methods

### 2.1 Search strategy

This study was designed and reported according to the PRISMA guidelines. A comprehensive literature search was conducted across four databases: PubMed, Embase, Cochrane Library, and Web of Science. The search was conducted from the inception of each database up to April 2025. The search strategy utilized a combination of MeSH terms and free-text keywords, including “Sepsis,” “Coagulation,” “mortality” and “prognosis,” with appropriate adjustments for each database’s specific syntax. Reference lists of the included studies were manually checked to identify any additional relevant studies.

### 2.2 Inclusion and exclusion criteria

Inclusion criteria: (1) Observational studies or randomized controlled trials (RCTs); (2) Participants diagnosed with sepsis; (3) Studies reporting at least one coagulation function parameter (such as APTT, D-Dimer, Fibrinogen, INR, PT) and its association with key clinical outcomes (such as mortality, organ dysfunction, ICU stay duration, or mechanical ventilation usage), with corresponding OR or AUC values provided. OR quantifies the association between coagulation markers and mortality risk, while AUC evaluates their overall prognostic discrimination.

Exclusion criteria: (1) Studies that are not observational studies or RCTs, such as reviews, commentaries, editorials, or non-research articles; (2) Studies that do not assess the relationship between coagulation function parameters and sepsis prognosis; (3) Studies with incomplete data that cannot be calculated from the provided information, and for which the authors could not be contacted for clarification, were excluded from the analysis.

### 2.3 Study selection and data extraction

Two reviewers independently screened the titles and abstracts of identified articles. Full-text reviews were conducted for potentially eligible studies, and data extraction was carried out independently by two reviewers. Extracted data included study design, sample size, patient characteristics, coagulation function parameters analyzed, and reported outcomes. In case of missing data, the authors were contacted for clarification. During study selection, any disagreements between the two independent reviewers were resolved through consensus discussion, with arbitration by a third senior investigator when necessary.

### 2.4 Risk of bias assessment

The risk of bias in the included studies was assessed using the PROBAST tool, which evaluates potential biases in participant selection, measurement of coagulation parameters, outcome assessment, and statistical analysis. It systematically evaluates potential biases across four key domains: participant selection, predictors, outcome assessment, and statistical analysis. Disagreements between reviewers were resolved through consensus or by consulting a third reviewer.

### 2.5 Statistical methods

Meta-analysis was performed using R software (version 4.3.3) with the “meta” and “metafor” packages. For predicted outcomes, the area under the curve (AUC) values were used, while for dichotomous outcomes, odds ratios (OR) were calculated. The results were expressed as odds ratios with 95% confidence intervals (CIs). Heterogeneity was assessed using the *I*^2^ statistic, with a random-effects model applied if *I*^2^ ≥ 50%, and a fixed-effect model used if *I*^2^ < 50%. Sensitivity analysis was performed to assess the robustness of the results.

## 3 Results

### 31 Literature search results and general characteristics

**FIGURE 1:**
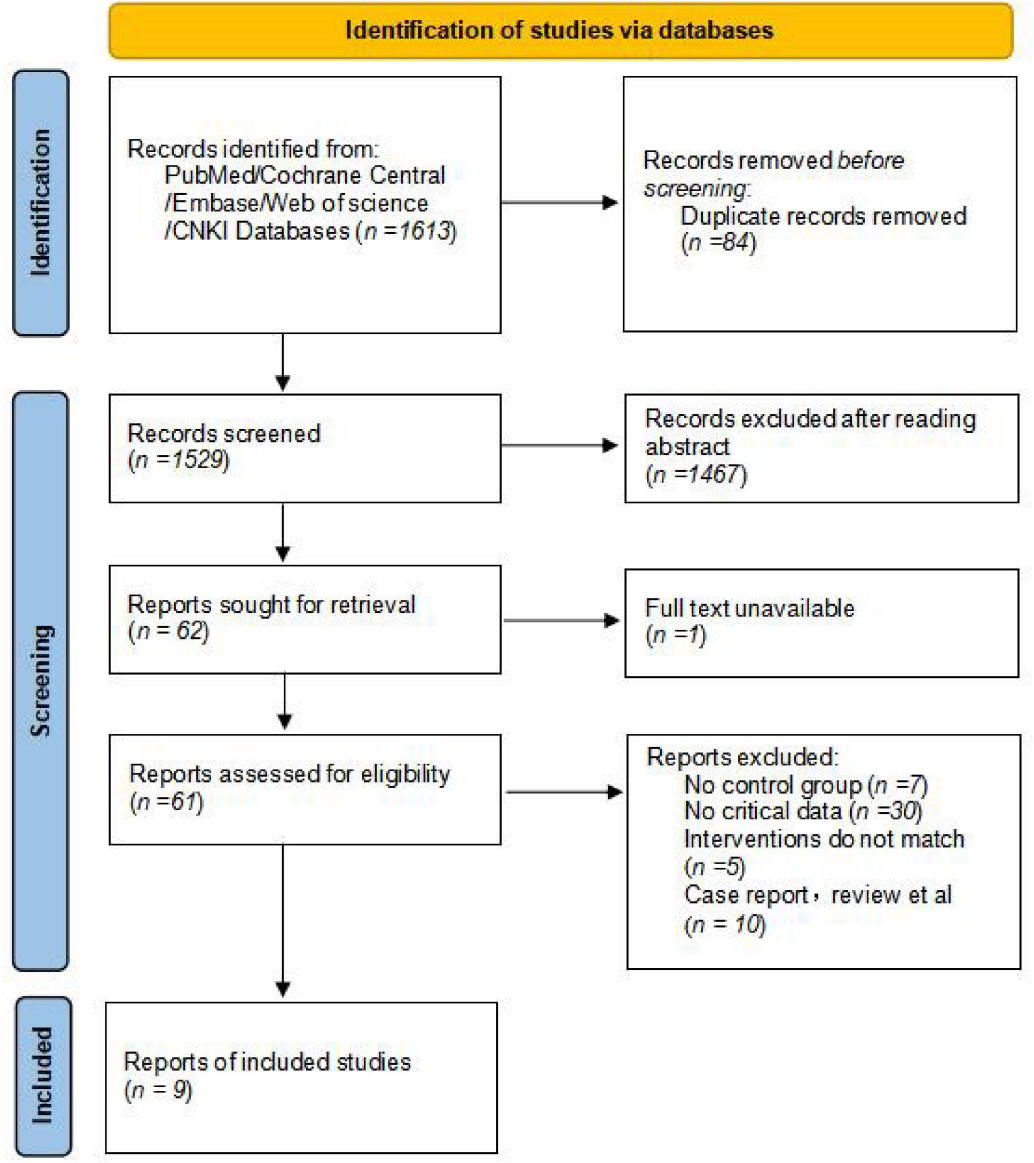
Flow chart.

A total of 1,613 relevant articles were identified through searches in domestic and international databases. After applying the inclusion and exclusion criteria, 9 studies (11–19) were included, involving a total of 1,954 patients. The literature screening flowchart is shown in Figure 1, and the basic characteristics of the included studies are presented in Table 1.

**TABLE 1:** Baseline characteristics.

First author	Publication year	Number of cases		Age (year)	
		Exposure group	Control group	Exposure group	Control group
Liu (17)	2021	17	49	77(63–84)	69(54–82)
Semeraro (19)	2019	89	89	70(62–77)	71(63–77)
Fu (13)	2023	44	94	70.56 ± 14.44	65.36 ± 16.91
Levi (16)	2020	191	194	63(55–74)	63(55–75)
Iba (14)	2018	419	30	77(73–82)	74(65–82)
Czempik (12)	2022	139	78	66(58–74)	67(56.5–57.2)
Lorente (18)	2022	80	134	62.7 ± 13.9	55.7 ± 15.4
Bui-Thi (11)	2023	91	70	69(60–81)	
Lemiale (15)	2022	64	82	65(57–72)	61(49–66)

### 3.2 Risk of bias assessment for the included studies

**FIGURE 2:**
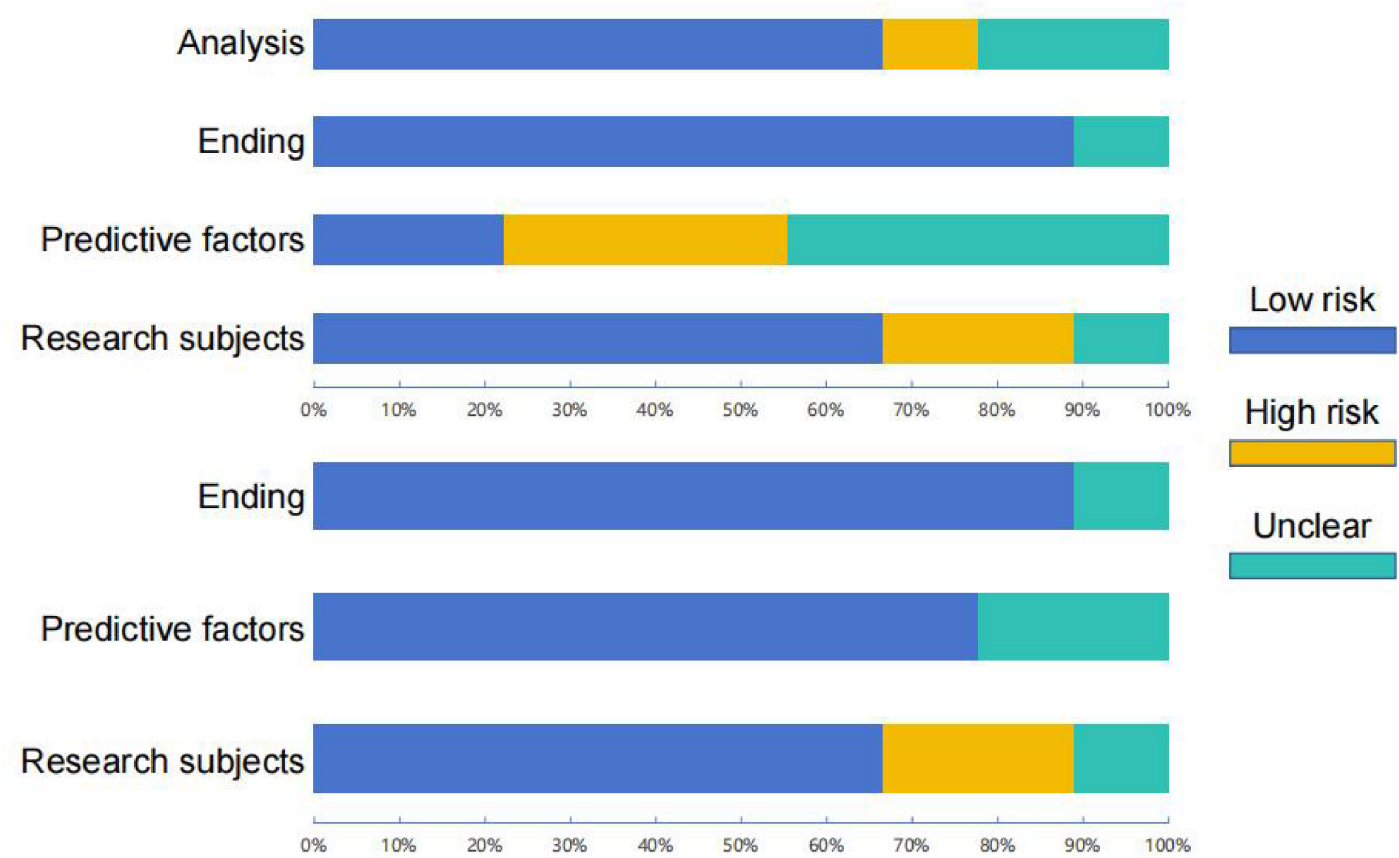
Risk of bias graphs.

In this meta-analysis, we assessed the risk of bias for 9 studies. Overall, most studies showed a low risk of bias in research subjects and ending. However, several studies had unclear information regarding analysis, predicted facto, which prevented the complete exclusion of bias risk in these areas (Table 2; Figure 2).

**TABLE 2:** Schematic table of PROBAST assessment results.

	Risk of bias				Applicability			Overall	
Study	Research subjects	Predictive factors	Ending	Analysis	Research subjects	Predictive factors	Ending	Risk of bias	Applicability
Liu	+	?	+	+	+	+	+	?	+
Semeraro	+	?	+	?	+	?	+	?	?
Fu	−	−	+	+	−	+	+	−	−
Levi	+	?	+	?	+	?	+	?	?
Iba	−	−	+	+	−	+	+	−	−
Czempik	+	−	?	−	+	+	?	−	?
Lorente	+	?	+	+	+	+	+	?	+
Bui-Thi	?	+	+	+	?	+	+	?	?
Lemiale	+	+	+	+	+	+	+	+	+

Low risk: +; High risk: −;Unclear: ?

### 3.3 APTT

**FIGURE 3:**
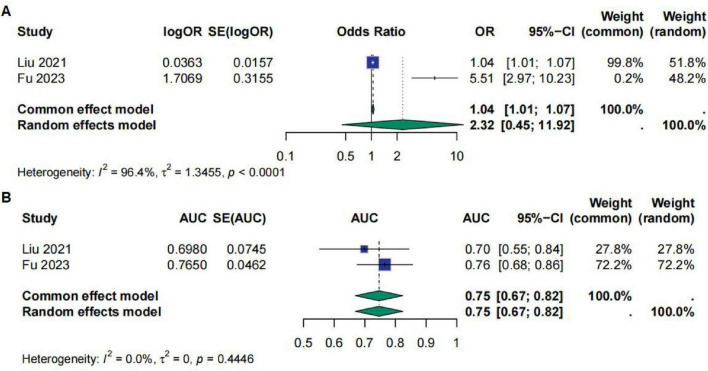
**(A)** Meta-analysis for OR of APTT. **(B)** Meta-analysis for AUC of APTT.

In the evaluation of APTT as a prognostic marker for sepsis patients, we included two studies. The meta-analysis of OR values showed that the OR for APTT in predicting sepsis prognosis was 2.32 [95% CI (0.45; 11.92)], which was not statistically significant. The heterogeneity test indicated *I*^2^ = 96.4%, τ^2^ = 1.3455, *p* < 0.0001, suggesting severe heterogeneity in the OR values of APTT across the studies. However, the pooled AUC result indicated an AUC value of 0.75 [95% CI (0.67; 0.82)] with no significant heterogeneity between studies, indicating that APTT has some value in predicting the prognosis of sepsis patients (Figure 3).

### 3.4 PT

**FIGURE 4:**
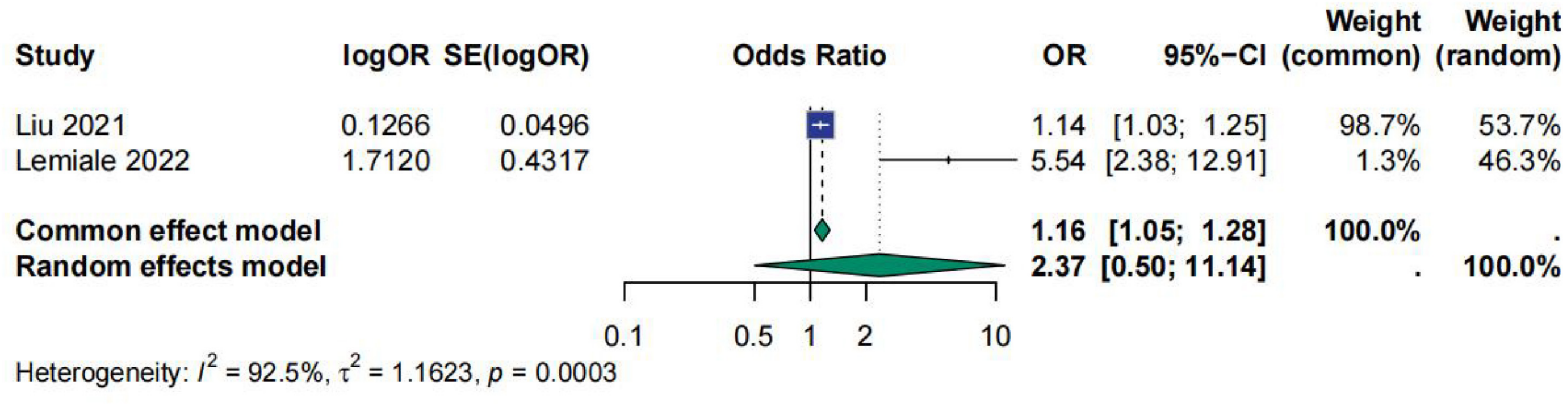
Meta-analysis for OR of PT.

A total of two studies involved PT, with *I*^2^ = 92.5%, τ^2^ = 1.1623, and *p* = 0.0003. Based on this, we selected a random-effects model for analysis. The results showed that the pooled effect size OR for PT was 2.37 [95% CI (0.50; 11.14)], indicating that PT has no significant correlation with sepsis prognosis and that its effectiveness in predicting the prognosis of sepsis patients varies considerably (Figure 4).

### 3.5 D-Dimer

**FIGURE 5:**
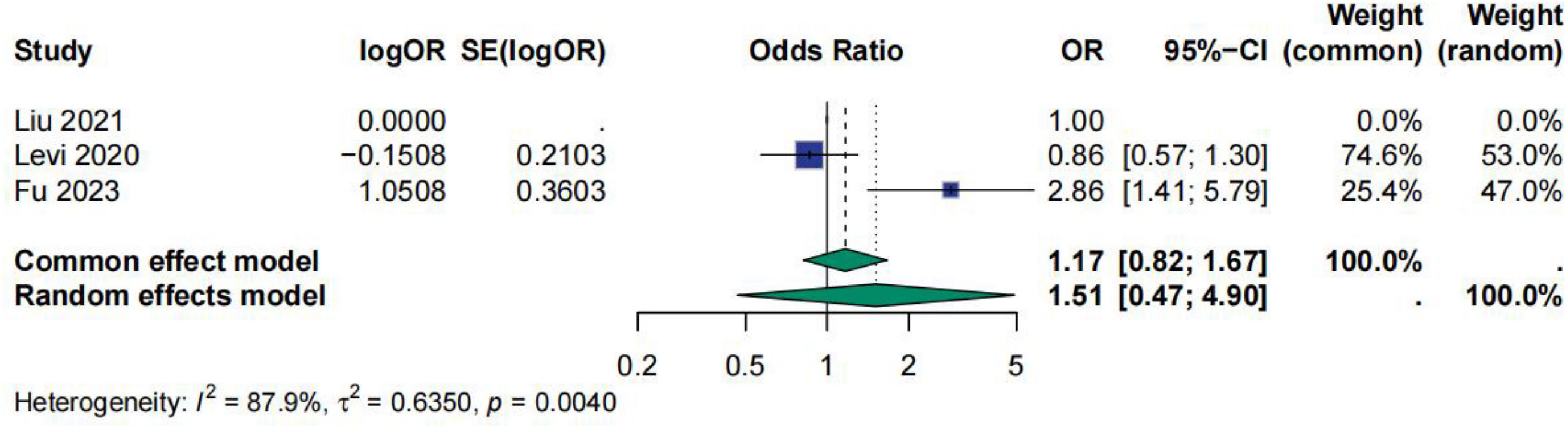
Meta-analysis for OR of D-Dimer.

For the studies on D-Dimer, a total of three studies were included. The meta-analysis results showed that the pooled effect size OR for D-Dimer was 1.51 [95% CI (0.47; 4.90)], and the heterogeneity test indicated *I*^2^ = 87.9%, τ^2^ = 0.6350, *p* = 0.0040. This suggests that D-Dimer has no significant correlation with sepsis prognosis and there is considerable heterogeneity among the studies. The role of D-dimer in predicting the prognosis of sepsis patients therefore remains highly uncertain (Figure 5).

### 3.6 Fibrinogen

**FIGURE 6:**
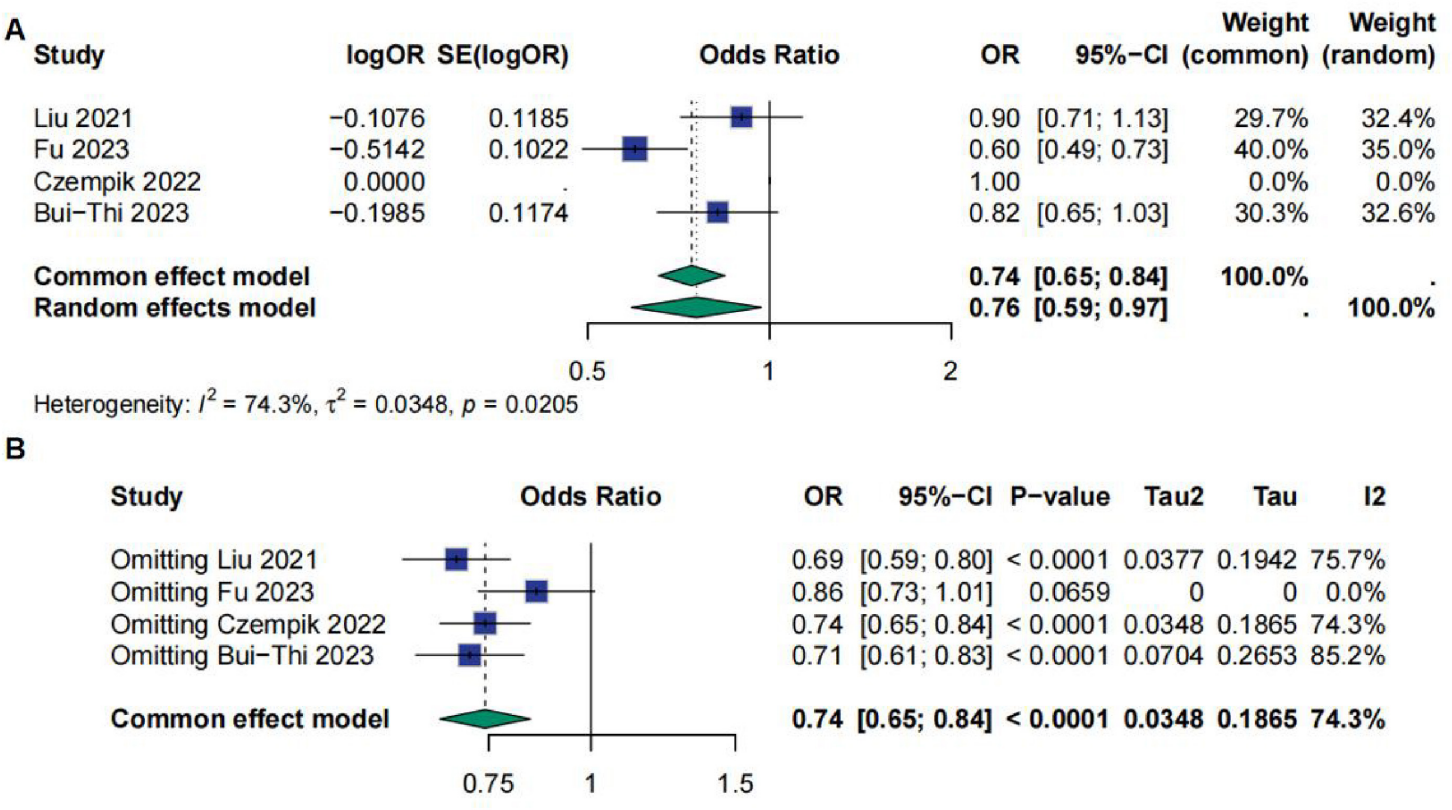
**(A)** Meta-analysis for OR of Fib. **(B)** Sensitive analysis for Fib.

A total of four studies involved Fib (fibrinogen). The meta-analysis results indicated that the OR for Fib was 0.76 [95% CI (0.59; 0.97)], suggesting a significant correlation between Fib and sepsis prognosis. However, the results showed high heterogeneity, with *I*^2^ = 74.3%, τ^2^ = 0.0348, and *p* = 0.0205. Sensitivity analysis demonstrated that after excluding the study by Fu et al., the results became non-significant (Figure 6).

### 3.7 INR

**FIGURE 7:**
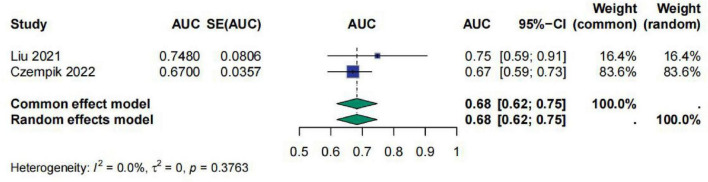
Meta-analysis for AUC of INR.

Regarding INR, a total of two studies evaluated the predictive efficacy of INR for sepsis prognosis using ROC curves. This study performed a meta-analysis on the AUC values from these studies. The meta-analysis results showed that the AUC value for INR was 0.68 [95% CI (0.62; 0.75)], indicating that INR has moderate predictive ability for sepsis prognosis. The heterogeneity test results indicated *I*^2^ = 0.0%, τ^2^ = 0, and *p* = 0.3763, suggesting no significant heterogeneity among the studies (Figure 7).

### 3.8 SOFA score

**FIGURE 8:**
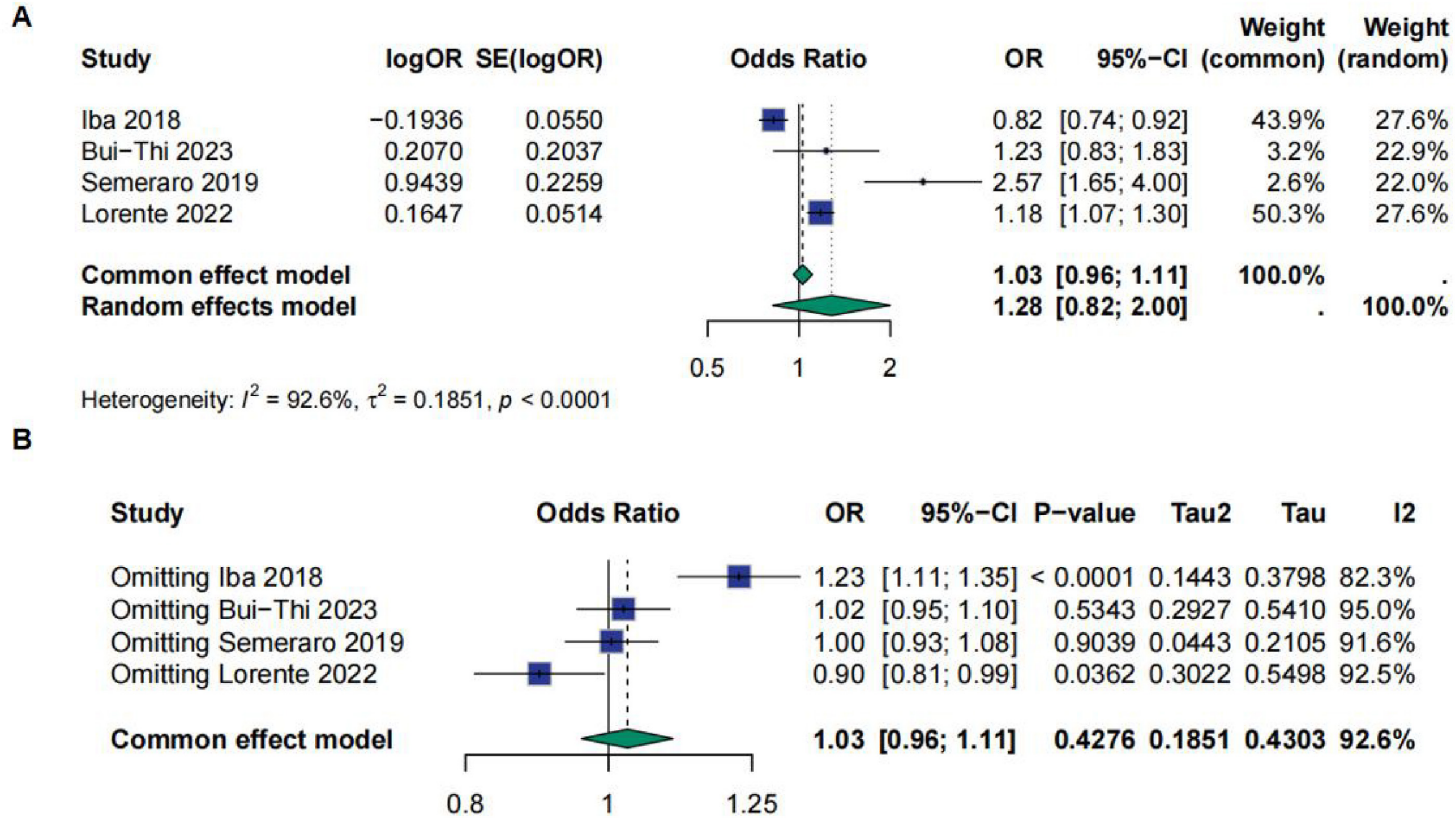
**(A)** Meta-analysis for OR of SOFA. **(B)** Sensitive analysis for SOFA.

**FIGURE 9:**
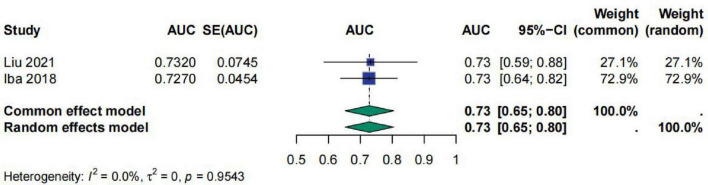
Meta-analysis for AUC of SOFA.

A total of four studies involved the SOFA score, with two of them reporting the area under the ROC curve for the SOFA score. The meta-analysis results showed that the OR value for the SOFA score was 1.28 [95% CI (0.82; 2.0)], indicating no significant correlation between the SOFA score and sepsis prognosis. However, the studies exhibited high heterogeneity, with *I*^2^ = 92.6%, τ^2^ = 0.1851, and *p* < 0.0001. Sensitivity analysis also indicated that the results were not robust (Figure 8). Additionally, a meta-analysis of the AUC for the SOFA score revealed an AUC of 0.75 [95% CI (0.65; 0.80)], suggesting that the SOFA score has good predictive ability for sepsis prognosis and shows no significant study heterogeneity (Figure 9).

## 4 Discussion

Sepsis is a systemic inflammatory response syndrome triggered by infection (2, 20), characterized by insidious onset, rapid progression, and high mortality. It can lead to multiple organ dysfunction and even death. Early identification and risk stratification are crucial for improving patient outcomes. In clinical practice, coagulation dysfunction represents a key pathological process in sepsis, with manifestations such as consumption of clotting factors, activation of the fibrinolytic system, and microthrombus formation (4). These changes may not only reflect the severity of illness but also hold potential prognostic value. Therefore, exploring the predictive utility of coagulation-related indicators and scoring systems for sepsis prognosis has important clinical implications.

This meta-analysis synthesized current evidence on the associations of APTT, PT, D-dimer, fibrinogen, INR, and SOFA scores with sepsis prognosis. The findings revealed notable variability in predictive performance across these indicators, with some demonstrating substantial heterogeneity between studies. This heterogeneity may be attributable to differences in patient characteristics, disease stages, treatment regimens, study designs, and laboratory testing standards. In addition, the definitions of outcome events varied among studies—some used 28-day mortality, while others focused on in-hospital death—which may have introduced effect estimate bias and compromised the stability of pooled results.

Although APTT did not demonstrate statistical significance in pooled effect estimates, ROC curve analyses indicated potential predictive value. This discrepancy may reflect the complex regulation of APTT in sepsis. APTT prolongation in critically ill patients does not solely reflect coagulation impairment; it can also be influenced by hypothermia, acidosis, anticoagulation therapy, and inflammatory mediators, none of which were systematically adjusted for in the original studies (21, 22), thereby contributing to inter-study variability. Basic research has suggested that APTT prolongation during the early phase of sepsis may result from interactions between inflammation and the endogenous coagulation system (23), and may not directly indicate adverse outcomes.

PT and INR are commonly used indicators of the extrinsic coagulation pathway, yet they showed divergent results. PT is heavily influenced by laboratory-specific testing standards and thus prone to measurement error and unit inconsistency (24), limiting cross-study comparability. In contrast, INR, which is standardized across reagents, demonstrated more consistent prognostic performance in this meta-analysis. This finding aligns with evidence from large prospective cohorts and suggests that INR may serve as a more stable prognostic marker in sepsis. Moreover, INR may reflect the interplay between hepatic synthetic function and systemic inflammation (25), thus possessing dual clinical relevance in the context of sepsis.

D-dimer, a marker of fibrinolytic system activation, is commonly elevated across a range of clinical conditions but showed limited prognostic utility in sepsis (26), with substantial inter-study heterogeneity. This aligns with ongoing debate regarding the predictive validity of D-dimer in sepsis. Although D-dimer levels increase in conditions such as disseminated intravascular coagulation (DIC), acute respiratory distress syndrome (ARDS), and deep vein thrombosis, its specificity in sepsis is limited (27). Some studies have shown that D-dimer is strongly influenced by inflammatory burden, hepatic impairment, and infection source, with non-specific elevations potentially masking its prognostic utility (28). Additionally, inconsistency in threshold definitions and timing of measurement across studies may further explain the lack of significant pooled effects.

Although fibrinogen demonstrated a potential protective effect in pooled analyses, it was also associated with high heterogeneity, and sensitivity analyses suggested strong dependence on individual studies. Fibrinogen acts as an acute-phase reactant and may indicate preserved immune and coagulation compensatory mechanisms when moderately elevated (29), thereby correlating with favorable outcomes. However, excessively high or persistently elevated fibrinogen levels may reflect dysregulated inflammation or endothelial injury, implying a potential biphasic prognostic role. To date, studies exploring the mechanistic role of fibrinogen in sepsis remain limited, and it remains unclear whether dynamic changes in fibrinogen are more prognostically informative than static levels. Further prospective research is needed to validate these findings.

Recent studies have emphasized that dynamic changes in coagulation biomarkers may more accurately reflect disease progression and treatment response than single measurements. For instance, serial monitoring of D-dimer and fibrinogen trends has been shown to predict the transition from compensated to overt disseminated intravascular coagulation (4), while persistent INR elevation or a progressive decline in fibrinogen has been associated with poor outcomes in septic patients receiving anticoagulant therapy (30). Similarly, time-dependent increases in APTT or PT during the disease course may indicate worsening coagulopathy and organ dysfunction, even when baseline levels are inconspicuous (31). These findings suggest that longitudinal assessment of coagulation markers, rather than static values alone, could provide deeper insight into the temporal dynamics of sepsis-associated coagulopathy. Therefore, incorporating serial biomarker trends into future studies may improve prognostic accuracy and facilitate earlier recognition of clinical deterioration. The clinical applicability of coagulation biomarkers is also noteworthy. INR and fibrinogen, which are rapidly obtainable in ICU settings, show potential for bedside risk stratification. INR may help identify patients with impaired coagulation reserve, while fibrinogen can complement existing severity assessments by reflecting the interaction between inflammation and compensatory coagulation responses. Incorporating these markers with SOFA or other clinical scores may enhance early prognostic evaluation. However, prospective studies are still needed to determine whether their integration into clinical pathways can improve decision-making and outcomes.

This study has several limitations. First, the number of included studies was relatively small, and some indicators such as APTT, PT, and INR were represented by only two studies, which lowers statistical power and reduces confidence in the pooled estimates. Second, some studies lacked complete raw data or only provided graphical results, potentially affecting data-extraction accuracy. Third, heterogeneity in clinical assessments, interventions, and outcome definitions limited comparability and generalizability. Fourth, potential publication bias may exist, since studies with non-significant or negative results are less likely to be published, possibly inflating reported associations. In addition, we focused on individual coagulation parameters without incorporating composite coagulation scoring systems, and subgroup analyses were not possible due to limited data. Future research should include larger, high-quality prospective studies to validate these findings.

Despite these limitations, this meta-analysis has several notable strengths. It provides a systematic synthesis of multiple coagulation-related indices using contemporary evidence and highlights the distinct prognostic contributions of each marker. The findings underscore the potential value of integrating coagulation parameters into sepsis risk assessment and point to promising avenues for clinical application. Future investigations should prioritize large-scale prospective cohorts, standardized measurement protocols, and dynamic monitoring frameworks to assess temporal biomarker trajectories. Moreover, combining coagulation markers with composite clinical scores or machine-learning–based predictive models may help refine individualized risk stratification and support precision management strategies in sepsis.

## Data Availability

The original contributions presented in the study are included in the article/supplementary material, further inquiries can be directed to the corresponding author.
